# Vaccination delays maedi-visna lentivirus infection in a naturally-infected sheep flock

**DOI:** 10.1186/1746-6148-9-16

**Published:** 2013-01-22

**Authors:** Margrét Gudnadóttir, Andreas Demosthenous, Theophanis Hadjisavvas

**Affiliations:** 1 Department of Microbiology, University of Iceland, Ármúli 1A, Reykjavík, 108, Iceland; 2 National Veterinary Services, Limassol, Cyprus; 3 Veterinary Laboratories, National Veterinary Services, Nicosia, Cyprus

**Keywords:** Lentivirus, Maedi-Visna, Vaccine-trial, Challenge by natural infection

## Abstract

**Background:**

The Maedi-Visna (MV) lentivirus causes two slowly progressive eventually fatal diseases of sheep, Maedi, a progressive interstitial pneumonia, and Visna, a progressive demyelinating disease of the central nervous system. Other lentiviruses also cause fatal slow infections in their natural hosts, e.g. the HIV virus in humans. Results of experimental vaccination against any lentivirus where vaccinees are challenged by natural routes, may therefore be of general interest. From 1991–1998 experiments with formalin-inactivated whole Maedi-Visna virus vaccine were carried out in the Department of Microbiology at the University of Iceland. Western Blot tests showed good immune response to all major proteins of the virus. When aluminium hydroxide was added to the vaccine all vaccinees developed neutralizing antibodies to the vaccine strain at titers 1/8 – 1/256. After housing 5 twin pairs, one twin in each pair vaccinated, the other unvaccinated, with infected sheep for 4 years, all the unvaccinated twins became infected, but only 2 of their vaccinated siblings as confirmed by virus cultivation experiments on tissues from their lungs spleens lymph nodes and choroid plexuses.

**Results:**

One twin in each of 40 female twin pairs, born into a Maedi-Visna-infected sheep flock and kept under natural farming conditions in Cyprus, was vaccinated at birth, 3 weeks and 3 months, with formalin-inactivated whole Maedi-Visna lentivirus vaccine adjuvanted with aluminium hydroxide. 17 mothers of the twins were seronegative, 13 seroconverting and 10 had old infection. Of 17 vaccinees born to seronegative mothers 9 were uninfected at 28 months, but only 2 of their unvaccinated siblings. Of 13 unvaccinated twins born to seroconverting mothers, 12 caught infection during their first 10 weeks, but only 4 of their vaccinated siblings. Vaccination had no effects on 10 vaccinees born to mothers with long-standing Maedi-Visna infections and broad andibody response at birth of their lambs.

**Conclusion:**

Compared with their unvaccinated siblings, natural infection was delayed in significant number of vaccinated twins born by seronegative and seroconverting mothers and vaccinated at birth, 3 weeks and 3 months with formalin inactivated whole MV vaccine adjuvanted with aluminium hydroxide. Maternal antibodies interfered with vaccination so early in life if the mother had old infection.

## Background

Maedi, progressive interstitial pneumonia, and Visna, progressive demyelinating disease of the central nervous system, are two serious, slowly progressive and eventually fatal diseases of sheep caused by the same lentivirus infection, the Maedi-Visna (MV) virus infection [1-3]. Other lentiviruses also cause fatal slow infections e.g. the HIV virus in humans. Experimental vaccination against any animal lentivirus may therefore be of general interest. From 1991–1998 experiments with formalin-inactivated whole Maedi-Visna virus vaccine were carried out in the Department of Microbiology at the University of Iceland [4,5]. The vaccinees were kept in isolation cubicles during the vaccination period. Western Blot (WB) tests on sera from the first 16 vaccinees showed good immune response to all major proteins of the virus, and when aluminium hydroxide was added to the vaccine they all developed neutralizing antibodies to the vaccine strain at titers 1/8 – 1/256 or higher. After booster injection of vaccine the WB bands became clearer and neutralizing titers rose [4].

Housing infected sheep with healthy flocks was the main mode of natural Maedi-Visna transmission from one farm to another in Iceland during the epidemic 1933–1952 [3]. This route of transmission was therefore chosen as the challenge in these experiments. The 16 fully vaccinated sheep and their controls were moved from their isolation cubicles to a sheep hut and housed there with 6 heavily infected sheep. After being housed with the infected sheep for 4 years, 5 unvaccinated twins in 5 twin pairs had become infected, but only 2 of their vaccinated siblings. These results were confirmed by virus cultivation experiments on tissues from spleens, lymph nodes, lungs and choroid plexus from all 10 twins [5]. After these experiments it was tempting to test the effects of this vaccine in a naturally-infected sheep flock. Infected flocks are not available in Iceland; the last one being eradicated by slaughter in 1965. Maedi-Visna infection is common in the Mediterranean region of Europe. The National Veterinary Services in Cyprus were introduced to these results, decided to participate in a field trial of the vaccine and found a place for the experiment on one very good sheep farm.

## Methods

### Vaccine strain of virus

Icelandic Maedi-Visna strain K796 was selected for preparation of vaccine, because long-term animal experiments carried out 1960–1975 had demonstrated that this strain was highly immunogenic and caused both Visna and Maedi in the experimental sheep [6]. Strain K796 was originally isolated from the brain of a symptomatic sheep in the 5^th^ brain-to-brain laboratory passage of natural Visna. In preparation for this vaccine trial in Cyprus, WB tests on a few sera from older sheep in the flock that had been selected to house the experiment tested positive against strain K796, indicating that related strains circulated in the flock.

### Vaccine preparation

Virus was grown in sheep choroid plexus tissue culture from 4 month-old Icelandic lambs. Cells were grown in tissue-culture flasks on a flat surface, (80cm^2^), in medium 199 with Earl’s salts (199E, Gibco, UK) and 20% Icelandic lamb serum. When the cell cultures were confluent the medium was changed to 199E with 2% lamb serum and the tissue cultures infected. Cytopathic lesions were at their maximum, 8–9 days after infection and then the tissue culture fluid was harvested, cell debris removed by centrifugation at 3332G for 40 minutes and the supernatant inactivated in formalin (Merk, Germany) 1:4000 at 37°C for 4–5 days. At the end of the inactivation period the fluid was divided into 200 ml aliquots, which were centrifuged at 22524G for 4 hrs. All supernatant fluid was then discarded and the pellet in each bottle resuspended in 2 ml. medium 199E without serum, thus concentrating the virus preparation 100x. In order to test for failure of inactivation, 2 ml. of concentrate from one pellet were inoculated into 2 bottles of tissue culture and incubated 5–6 weeks with frequent microscopic examinations for cytopathic lesions. When this control was finished, sterile aluminium hydroxide (Fisher Scientific Company, USA. E&A Tested Purity Reagent) powder, 5 mg/ml, was added to the vaccine. After mild shaking twice a day for one week the vaccine was ready for use.

### Vaccinees

The vaccinees were one twin lamb in each of 40 female twin pairs born to mothers of the Chios breed of sheep into an infected flock of about 700 adult sheep on one well maintained sheep farm in Cyprus. The other twin served as an unvaccinated control throughout the experiment. Mothers of these twins were selected according to results of AGID screening tests on all pregnant ewes in the flock a month before birth of these lambs: 30 mothers seronegative and, for comparison, 10 mothers seropositive in the AGID test. Blood samples were collected from mothers and both twins at birth of each twin pair when one twin in each pair was vaccinated. Blood samples from both twins in each pair were also collected 3 weeks after the first, second and third vaccination, then again 6 weeks after the 3^rd^ vaccination and thereafter at 5–7 months intervals during the next 2 years. Serum was harvested from all blood samples and frozen at – 20°C. The original plan for this experiment was to vaccinate one twin lamb in 30 female twin pairs born into a naturally infected sheep flock by seronegative mothers and, for comparison, one twin in 10 pairs born by seropositive mothers. When sera were recollected from the mothers at birth of their lambs, 13 of the 30 mothers negative in AGID tests 4–5 weeks earlier were positive in WB tests against the vaccine strain K796. All the 10 mothers previously positive in the AGID tests were positive in the WB tests. In accordance with different immunological experience of the mothers the vaccinees were divided into 3 groups:

Group 1) 17 twin pairs born by mothers seronegative both in AGID and WB tests.

Group 2) 13 twin pairs born by mothers negative in AGID, but positive in WB tests.

Group 3) 10 twin pairs born by mothers positive both in AGID and WB tests.

The flock was kept and fed on the sheep farm under natural farming conditions throughout the experiment. According to farming traditions, newborn lambs are kept with their mothers for their first 35 days of life, then taken away from the mothers and moved to the main flock. This applied to the experimental lambs as well as all other lambs born into this flock.

This experiment was approved of the Chief Veterinary Officer in Cyprus, who also was the Director of the Department of Veterinary Services at the Ministry of Agriculture, Natural Resources and Environment. The Ministry supported this work by a grant for maintenance of the experimental sheep, as two branches of the National Veterinary Services, the Central Virus Laboratory in Nicosia and the National Veterinary Services in Limassol, were major participants in this work.

### Vaccination

Vaccine, 1 ml per dose, was given at the birth of the vaccinee, a second injection 3 weeks later, then a third injection when the lambs were 3 months old. There were no later boosters. Vaccine was injected intramuscularly with no side effects.

### Diagnostic laboratory work

The AGID screening tests, then used for diagnostic work at the Virus Laboratory of the National Veterinary Services in Cyprus were performed on over 600 pregnant ewes on the farm during the last month of their pregnancy in order to select mothers for the vaccinees. Commercially available test plates (Weybridge Laboratories, England) were used in this work and the tests were performed by senior technicians according to instructions for use of the plates. Methods for Western Blots (WB) and Complement Fixation (CF) tests done in Iceland have been previously described and compared [7,8]. The CF test was chosen for laboratory diagnosis of infection in vaccinees and their unvaccinated siblings because it is a reliable diagnostic test [3,6,9]. It is comparable with the WB test in time of antibody response after infection [8]. Good CF antigens, that is high titer untreated tissue culture fluid, could be prepared from local Maedi-Visna strains isolated from other sheep in the flock. Their primary isolation in choroid plexus cells from Icelandic lambs was time consuming and their growth low and slow as compared with K796. Serial 2 fold dilutions of sera were prepared and mixed with antigen and 4 units of complement (Guinea Pig Complement, Harlan Sera Lab, UK). This mixture was kept overnight at + 4°C, the hemolytic system added next morning and reading done 3–4 hours later. Four-fold or larger difference in titer is significant in a CF test [9].

## Results

### Immunity in the sheep flock

About 40% of the 600 pregnant ewes tested in AGID tests were positive. Later in this study we found that 13 of the 30 mothers negative in the AGID test were positive in Western Blot (WB) tests against the vaccine strain of virus. All the 10 mothers previously positive in the AGID test, also tested positive in the WB test. If these results of the WB tests hold for the whole flock, it is heavily contaminated with Maedi-Visna virus infection. No illness was found in the flock during the time of this trial and the sheep thrived well.

### Strain difference

Local strains used as CF antigens showed 8-16-fold higher titers than K796 in sera from the infected sheep. Table 1 shows comparison of CF titers against three CF antigens, two from local strains and the vaccine strain K796.

**Table 1:** Comparison of CF titers against 3 Maedi-Visna strains, vaccine strain K796 and 2 local strains 10B and 7W from 2 sheep of the flock in Cyprus

**Titer of serum from one sheep infected intrapulmonarily with 300.000 TCID 50 of virus K796**	**Virus strains**		
	**K796**	**10B**	**7W**
**Serum 13 months after infection with K796**	128	16	8
**Serum 3 years after infection with K796**	256	32	16
**Titer of serum from one naturally infected sheep in Cyprus**	16	128	128

* In these CF tests serial 2 fold dilutions of sera ranging from dilutions 1/4 - 1/512 were tested against the 3 strains. Four-fold or larger difference in titer is significant.

### Results of vaccination

The results of the vaccination varied according to the immune status of the mothers at birth of their twins. Therefore, the twin pairs were divided into 3 groups as previously described. Table 2 shows the results of the vaccination in all 3 groups.

**Table 2:** Number of infected lambs in the 3 groups

**Age in months:**	**2-4**	**5**	**11**	**16**	**23**	**28**
**Group 1**						
**Vaccinated**	**0**	**0**	**1**	**6**	**8**	**8 (47%)**
**Unvaccinated**	**0**	**0**	**2**	**9**	**14**	**15 (88%)**
**Group 2**						
**Vaccinated**	**4**	**5**	**11**	**13**	**13**	**13**
**Unvaccinated**	**12**	**12**	**12**	**13**	**13**	**13**
**Group 3**						
**Vaccinated**	**0**	**0**	**0**	**At 18 months 9 pairs alive.**		
**Unvaccinated**	**0**	**0**	**0**	**In 4 pairs both lambs seronegative.**		
				**In 3 pairs both lambs infected.**		
				**In 2 pairs one lamb negative one vaccinee and one unvaccinated.**		

Table 2 shows that during the first half of their first year there was no seroconvertion detectable in any lamb in Group 1. At 11 months 3 lambs had seroconverted, 2 unvaccinated and 1 vaccinee. At 16 months of age, 15 lambs had caught infection: 9 unvaccinated and 6 vaccinees. At the end of the second year, 14 of 17 unvaccinated lambs had seroconverted but only 8 vaccinees. At 28 months 9 vaccinees were still seronegative, but only 2 of their unvaccinated siblings. The difference is significant (P = 0.025; Fisher’s Exact Test).

Table 2 also shows that during their first 2 months of life 12 of the 13 unvaccinated twins in Group 2 became infected and seroconverted, but only 4 of their vaccinated siblings, and the 5th one at 5 months of age. This difference at five months is significant (P = 0.01; Fisher’s Exact Test). Late in their first year of life, 6 of the vaccinated twins seroconverted and the last 2 early in their second year. At 16 months of age, all 26 lambs in this group were infected. When the vaccinees are compared with their unvaccinated siblings, with both lambs suckling milk from the same mother their first 35 days, vaccination is the most likely reason for the delay of milk born infection in the vaccinated twins.

Vaccination of the 10 twins in Group 3 was unsuccessful. There was no infection detected in any twin in this group during their first year of life, in contrast with the experience in Group 2. Of 9 twin pairs in Group 3, alive at 18 months after their first vaccination, both lambs in 4 pairs were seronegative, both lambs in 3 pairs seropositive and one lamb seropositive in 2 pairs, a vaccinee in one pair, and unvaccinated lamb in the other. It is probably not possible to vaccinate newborn or very young lambs if they are loaded with maternal antibodies following long-standing infection of the mother.

## Discussion

### Adverse reactions

The vaccine was well tolerated, and there were no adverse reactions, neither swelling after the injections nor systemic reactions. During the earlier experiments with this vaccine in Iceland the experience was the same [4].

### Antibody response

The antibody response to this vaccine was previously studied in seronegative Icelandic sheep kept in isolation, in spite of the fact that Iceland has been free of Maedi-Visna infection since 1965 [4]. In this field trial we did not test for antibody response after vaccination, because the vaccinees were exposed to natural infection from their environment every day after their birth, and therefore it was impossible to be certain whether they were responding to the vaccination or early natural infection from their environment. For the immune response to the vaccine we therefore refer to the studies done in isolation of the 16 seronegative Icelandic vaccinees, mentioned here in the Background section [4].

### Protection

As mentioned here in the Background section, a small experiment on 5 twin-pairs, one twin in each pair vaccinated and the other an unvaccinated control, showed, that after 4 years of close contact with sheep infected with the vaccine strain of virus all the unvaccinated twins had seroconverted, but only 2 of their vaccinated siblings. Cultivation of virus from lungs, spleens, hilus lymph nodes and choroid plexuses from all 10 twins confirmed these results. The 3 seronegative twins tested were also negative in all the tissue culture experiments [5]. This type of work can only be done at laboratory conditions, not at the natural conditions on an infected farm. In slow infections were you count the incubation period in years without clinical signs, you have to select the indirect method of antibody response as the most reliable method to separate real infection from contamination of specimens taken at field work. In this field trial the results shown in Table 2 for Group 1 at 28 months remind of the results found in the earlier twin experiment in Iceland and may mean protection, although our methods did not directly prove it. Therefore we prefer to call our findings in this field trial a delay of infection in the vaccinees which responded to the vaccination.

### Methods to detect infection in vaccinees and their unvaccinated controls

Those who are familiar with the diagnostic work on the HIV lentivirus infection in humans know, that positive ELISA test is not a reliable diagnostic test for HIV infection and must be confirmed by a more reliable test, e.g. Western Blot (WB) test on the ELISA positive serum. In most of these cases the ELISA test is false positive. On these grounds we selected another and more reliable test for this field trial.

In the HIV laboratory of the Department of Microbiology, University of Iceland, we developed Western Blot technique for Maedi-Visna and compared it with the CF test we had previously developed and used extensively during the eradication of the disease from Iceland [7,8]. The comparison with the WB test showed that the CF test became positive at the same time as the WB in all cases tested [8]. Therefore, the CF test, simple and reliable in diagnostic work on many human virus diseases became the test for natural infection in this field trial instead of the cumbersome Western Blot technique. If you compare the vaccinees with their unvaccinated controls as shown in Groups 1 and 2 in Table 2, you see that both the unvaccinated control twins and the vaccinated twins that did not respond to the vaccination became positive in the CF test at the same period of time. If you look at Group 1 only, you see that it is during the 2^nd^ year of the experiment that they develop antibodies if they are infected from the outside environment, but earlier if the infection is milk-born as in Group 2 in Table 2. If you look at Group 1 in Table 2 early in the 3^rd^ year of the experiment you see that only 2 unvaccinated sheep in the control group are seronegative, but 9 of their vaccinated siblings are still seronegative 2 years after their vaccination program ended.

### Boosters of vaccine

In this experiment no “boosters” of vaccine were given to vaccinees after 3 months of age. Infections of lambs in Group 3 are apparently not milk born infections, although their mothers were seropositive both in AGID and WB tests. This is in contrast to the experience among unvaccinated twins born by seroconverting mothers in Group 2. The lambs in Group 3 might have benefited from vaccination after 6 months of age, when passive maternal antibodies should no longer be interfering with the vaccination. Boosters late in the first year might also have prolonged the period of delay in those vaccineees in Groups 1 and 2, which responded to the vaccination. Unsuccessful vaccination in Group 3 is most likely caused by interference of passive maternal antibodies with vaccine given so early in life, a common finding if children are vaccinated during their first months of life.

### Clinical maedi-visna disease

The infected sheep flock, in which this experiment was conducted, was heavily contaminated with Maedi-Visna virus as judged by the tests on the mothers of the vaccinees and the rate and time of infection of the unvaccinated twins in Groups 1 and 2. Yet, there was no clinical illness in this flock and the sheep thrived well, although they were infected. Clinical Maedi-Visna is very rare in Cyprus, although the flocks are infected. The Icelandic experience from the importation of Maedi-Visna in 1933 until its total eradication in 1965, was different. There, clinical Maedi-Visna was disastrous in good sheep farming areas, culminating in the eradication by slaughter of all the infected flocks [3].

### Strain difference

The virus strains isolated from infected sheep in the flock housing this experiment were very slowly growing and it took many passages in tissue culture to obtain working titers of these strains. In spite of the obvious strain difference between the circulating virus strains isolated from the flock and the old Icelandic vaccine strain K796 (Table 1), monovalent vaccine made by inactivation of whole virus K796 apparently delayed infection in more than half of the number of vaccinees in Groups 1 and 2, when compared with their unvaccinated siblings living in the same infected environment and suckling the same mothers.

### Vaccine preparation and methods of challenge

More than twenty years ago two groups of workers, a multinational Scandinavian group and an American group, reported unsuccessful attempts at vaccinating sheep with inactivated Maedi-Visna and ovine progressive pneumonia (OPP) vaccines [10-13]. Both groups used methods very different from those used in the work described here. They used different vaccine strains of virus, different methods of virus inactivation and concentration, different adjuvants, if they were used, and last but not least different methods to challenge their results. Both groups used high-titer live virus to test the results of their vaccination. The Scandinavian group gave to their vaccinees and controls live virus by the respiratory route, but the American group injected large amounts of high-titer, live virus intravenously. Neither group had found neutralizing antibodies in their vaccinees, in contrast with the previous Icelandic experience with this vaccine [4]. The question arises as to whether it will ever be possible to determine if a lentivirus vaccine is working, by inoculating high titer of live virus directly in to the circulation of the vaccinee, where susceptible living cells, such as macrophages or T-cells, are located and may become infected. Such cells may carry the virus to distant organs, such as lymph nodes and lungs, where it can further multiply. Those who work with lentiviruses need to develop more natural challenge methods, although it takes time. Therefore, it needs to be remembered that a challenge by injecting a high titer of live virus is not comparable with the natural routes in an infected sheep flock.

### Inactivation

Through 19 years of work with this vaccine, all attempts to grow virus from concentrates of vaccine following inactivation of virus in tissue culture fluid have turned out negative. This may be relevant to those who have problems with other lentivirus vaccines and are against the idea of using whole inactivated virus in their experiments. It is, apparently, possible to treat a lentivirus by those conventional methods, that are used to produce good inactivated vaccines against poliovirus, hepatitis-A virus and influenza virus.

## Conclusion

The results of vaccination in Groups 1 and 2 seem to indicate that natural Maedi-Visna infection in a contaminated environment can be delayed, and in some cases possibly prevented, by conventional vaccination with formalin-inactivated whole virus vaccine adjuvanted with aluminium hydroxide.

## Competing interests

The authors declare that they have no competing interests.

## Authors’ contributions

All authors contributed equally to this work. MG prepared the vaccine and did all the laboratory work done in Iceland except the Western Blot tests. TH did all administrative work and design concerning this field trial in Cyprus and directed the virus laboratory work done there. AD did the veterinary work on the experimental sheep. He vaccinated the lambs and bled both vaccines and their unvaccinated siblings at regular intervals during the whole experimental period. He kept excellent record of these procedures and the health of the flock. All authors have read an approved this final copy of the manuscript.
